# Intensified Adjuvant Treatment of Prostate Carcinoma: Feasibility Analysis of a Phase I/II Trial

**DOI:** 10.1155/2014/480725

**Published:** 2014-06-30

**Authors:** Giovanna Mantini, Sergio Fersino, Anna Rita Alitto, Vincenzo Frascino, Mariangela Massaccesi, Bruno Fionda, Vincenzo Iorio, Stefano Luzi, Mario Balducci, Gian Carlo Mattiucci, Francesco Di Nardo, Antonio De Belvis, Alessio Giuseppe Morganti, Vincenzo Valentini

**Affiliations:** ^1^Unità Operativa di Radioterapia, Dipartimento di Bio-Immagini e Scienze Radiologiche, Università Cattolica del Sacro Cuore, Policlinico Gemelli, Largo A. Gemelli 8, 00168 Roma, Italy; ^2^Unità Operativa di Radioterapia, Fondazione di Ricerca e Cura “Giovanni Paolo II”, Università Cattolica del Sacro Cuore, Crt. Tappino 35, 86100 Campobasso, Italy; ^3^Dipartimento di Diagnostica per Immagini e Radioterapia, Policlinico Federico II, Via Pansini 5, 80131 Napoli, Italy; ^4^Istituto di Igiene e Medicina Preventiva, Università Cattolica del Sacro Cuore, Policlinico Gemelli, Largo A. Gemelli 8, 00168 Roma, Italy

## Abstract

*Purpose*. To perform a preliminary feasibility acute and late toxicity evaluation of an intensified and modulated adjuvant treatment in prostate cancer (PCa) patients after radical prostatectomy.* Material and Methods*. A phase I/II has been designed. Eligible patients were 79 years old or younger, with an ECOG of 0–2, previously untreated, histologically proven prostate adenocarcinoma with no distant metastases, pT2–4 N0-1, and with at least one of the following risk factors: capsular perforation, positive surgical margins, and seminal vesicle invasion. All patients received a minimum dose on tumor bed of 64.8 Gy, or higher dose (70.2 Gy; 85.4%), according to the pathological stage, pelvic lymph nodes irradiation (57.7%), and/or hormonal therapy (69.1%).* Results*. 123 patients were enrolled and completed the planned treatment, with good tolerance. Median follow-up was 50.6 months. Grade 3 acute toxicity was only 2.4% and 3.3% for genitourinary (GU) and gastrointestinal (GI) tract, respectively. No patient had late grade 3 GI toxicity, and the GU grade 3 toxicity incidence was 5.8% at 5 years. 5-year BDSF was 90.2%.* Conclusions*. A modulated and intensified adjuvant treatment in PCa was feasible in this trial. A further period of observation can provide a complete assessment of late toxicity and confirm the BDSF positive results.

## 1. Introduction

The incidence rates of prostatic carcinoma (PCa) increased in nearly all countries except in a few high-income countries. In contrast, the increase in PCa mortality rates mainly occurred in lower-resource settings, with declines largely confined to high-resource countries [1].

Radical prostatectomy (RP) is a common initial treatment for PCa. However, depending on tumor stage, 15–60% of patients develop a rise in PSA following radical prostatectomy [2]. Radiotherapy (RT) to the prostate bed has been used both adjuvantly and for salvage. There continues to be an active debate regarding when radiation should be administered, although 3 recent randomized trials show a consistent improvement in biochemical disease-free survival (BDFS) when adjuvant radiotherapy is administered as compared with radical prostatectomy alone [3–5]. Furthermore, RP, followed by postoperative RT in selected “high risk” patients, can be considered a treatment policy alternative to full radiation treatment for cure.

Based on a systematic review, adjuvant RT after RP improves overall survival and reduces the rate of distant metastases with longer follow-up and at 5 and 10 years it improves local control and reduces the risk of biochemical failure [6]. Because the morbidity of postoperative radiotherapy is relatively low, when pathologic high risk factors are present adjuvant radiotherapy is recommended by international guidelines [7, 8].

The EORTC-22911 trial was the first randomized clinical trial to demonstrate the advantage in terms of BDFS achievable by adjuvant RT [9]. However, even in patients receiving adjuvant RT the trial showed a 5-year biochemical failure rate higher than 25%. In that study, RT was administered only on the prostate bed with a total dose of 60 Gy. In order to improve these results in our centers a phase I/II has been designed to assess the possible impact of both modulated and intensified adjuvant treatment.

This study included the use of a higher dose in case of positive resection margins and/or perineural infiltration, considering the greater risk of failure in these patient categories [10, 11]. In addition, the dose given to the prostate bed was superior to that used in the EORTC study, based on preliminary data showing a better clinical outcome by means of doses higher than 61.5 Gy [12]. Moreover, whereas the presence of occult pelvic lymph node involvement may explain the failure of treatments targeted only to the prostate bed [13], elective irradiation of pelvic lymph nodes (ENI) was planned in high risk patients. Finally, on the basis of some evidence on the possibility of improving the results of postoperative RT by means of adjuvant hormonal therapy (AHT) [14, 15], the study included the use of AHT in patients with increased risk of treatment failure.

However, there is no evidence on the tolerability of an intensified adjuvant treatment as that provided in this study. The use of doses higher than those tested in randomized trials and the use of ENI can obviously worsen treatment tolerability. In addition, an increased RT-induced toxicity in patients undergoing pelvic surgery has been demonstrated [16]. Finally, some studies suggest that even the use of AHT may increase RT-induced toxicity [17, 18]. On the basis of this background, the aim of this analysis is to perform a preliminary feasibility evaluation of an intensified adjuvant treatment in terms of acute and late toxicity.

## 2. Material and Methods

### 2.1. Study Objectives

The primary study end point was biochemical disease-free survival, defined as the time from RP to first evidence of biochemical relapse. In particular, the primary objective of the study was to demonstrate an increase of 5-year BDSF from 75% to 90%. Biochemical relapse was defined as a PSA level exceeding 0.2 ng/mL after enrollment for those with a postsurgical PSA level of 0.2 ng/mL or lower and as two consecutive PSA increases for patients with a postsurgical PSA level of >0.2. Secondary outcomes included acute and late toxicity, local control, and metastasis-free survival, defined as the first evidence of any pelvic recurrence or extrapelvic recurrence of disease, respectively. Patients without the event of interest were censored at their last contact date (last PSA assessment date for PSA relapse).

### 2.2. Study Design

A phase I/II study was planned. Prior data [9] indicated that the success rate (5-year BDFS) among controls is around 0.75. If the true success rate for experimental subjects is 0.90, we would need to study 100 experimental subjects to be able to reject the null hypothesis that the success rates for experimental and control subjects are equal with probability (power) 0.8. Type I error probability associated with this test of this null hypothesis is 0.05. We used an uncorrected chi-squared statistic to evaluate this null hypothesis. Some overrecruitment was planned to allow for a continuous drop-out process of up to 20% during the follow-up period.

### 2.3. Inclusion Criteria

Eligible patients were 79 years old or younger, with an ECOG performance status of 0–2 and previously untreated, histologically proven adenocarcinoma of the prostate with no known distant metastases, and pathological stage pT2–4 N0-1, with at least one of the following risk factors: capsular perforation, positive surgical margins, or seminal vesicle invasion. A pelvic lymphadenectomy and an undetectable PSA level after RP were not required. Patients who underwent salvage RT were excluded from this analysis. Patients must have had evidence of adequate bone marrow and liver function. Previous radiotherapy or chemotherapy for prostate cancer was not allowed. Patients must not have had intraoperative rectal injury, persistent urinary extravasation, or pelvic infection. Tumor stage was determined according to the 1997 International Union Against Cancer criteria [19]. Before enrollment, all patients underwent pre- and postoperative PSA test, bone scan, CT scan or MRI of abdomen-pelvis, and chest radiography.

### 2.4. Radiotherapy

Simulation and treatment were performed in prone position using the up-down table (UDT), a special device aimed at reducing small bowel volume in the treatment field [20]. Patients were instructed to achieve stable conditions of bladder and rectal filling. Before CT simulation and before each therapy fraction patients were invited to (1) empty the bladder 2 hours prior to the procedure and drink 2 glasses of water right after and to (2) empty the bowel over the 2 hours prior to the procedure. RT was planned based on CT simulation performed after oral administration of contrast with 5 mm apart slices. Clinical target volume (CTV) definition was performed as follows: in the CTV1 the prostatic area with the sites occupied before surgery by the prostate and seminal vesicles was included. The lower margin of CTV1 was set at the cranial extremity of cavernous bulbs. The upper limit was defined based on the cranial extremity of seminal vesicles evaluated on preoperative CT or MRI. In the CTV2, obturator, external and internal iliac, and presacral (above S2-S3) to the sacral promontory were included. The planning target volume 1 (PTV1) was obtained by adding to the CTV1 a 5 mm margin posteriorly and an 8 mm margin in all other directions. The PTV2 was obtained by adding to the CTV2 an 8 mm margin in all directions. Conformal 3D plans were obtained with box technique and 6-beam technique for PTV2 and PTV1, respectively. Beams ≥ 10 MV collimated with standard multileaf collimators (2 × 40 leaves, width 1 cm at the isocenter) were used. The dose was specified according to the guidelines of the International Commission on Radiation Units [21]. Treatment was provided once a day, 5 days a week. Depending on tumor characteristics (Table 1), prescribed doses were the following:

1. pelvic node irradiation (45 Gy; 1.8 Gy/fraction) followed by boost on the prostate bed (19.8–25.2 Gy; 1.8 Gy/fraction; total dose: 64.8–70.2 Gy) or
2. exclusive prostate area irradiation (64.8−70.2 Gy; 1.8 Gy/fraction).

### 2.5. Adjuvant Hormonal Therapy

AHT was prescribed as indicated in Table 1. AHT was started simultaneously with the start of postoperative RT. The duration of ART was 6 months or 24 months depending on the risk category (Table 1). Patients were informed about the different characteristics and side effects of available hormonal therapies. It was then allowed to choose between the following adjuvant hormonal treatments:

1. LH-RH analogue: leuprorelin, 3.75 every month or 11.25 mg every 3 months, intramuscularly, or
2. antiandrogen agent: bicalutamide, 150 mg per day.

### 2.6. Statistical Analysis

A descriptive analysis of the sample was carried out by means of mean and standard deviation (SD) for continuous variables and absolute and relative frequencies for qualitative ones. All patients were analyzed for radiotherapy toxicity. Toxicity was monitored weekly during radiotherapy. Clinical examinations including digital rectal examinations and PSA tests were done every 3 months for 2 years, then every 6 months until the end of the fifth year, and then every year. Additional staging studies (e.g., bone scans) were performed as clinically indicated. Acute adverse effects of RT were scored according to the Radiation Therapy Oncology Group (RTOG) scale [23]. The Late Radiation Morbidity Scoring Scheme of the RTOG/European Organization for Research and Treatment of Cancer (EORTC) was used to assess late toxicity [23]. Differences in toxicity were studied by means of chi-squared and Fisher's exact tests. Analyzed variables were age at diagnosis (stratified as below or equal to 65 and higher than 65), ENI (yes versus no), prostate bed dose (64.8 versus 70.2), and AHT (no versus antiandrogen versus LH-RH analogue). Even a BDFS analysis was performed. The analysis was performed using SPSS software version 12.0 for Windows. Statistical significance level was set at *P* = 0.05.

### 2.7. Ethical Issues

Written informed consent was obtained from all patients. The study was approved by the institutional review boards of the participating institutions.

## 3. Results

One hundred twenty-three patients were enrolled in the study, and they completed the planned adjuvant treatment. Median follow-up was 50.6 months (interquartile range, 29.2–80.0 months). Characteristics of study participants and treatment characteristics are displayed in Table 2. Ninety-one patients were treated at the Catholic University of Rome and 32 at the “Fondazione Giovanni Paolo II” in Campobasso. Eighteen patients had a pathological stage pN1 disease (14.6%), 4 patients had a pathological stage pT4 tumor (3.3%), and 9 patients had a postoperative PSA level > 0.2 ng/mL (7.3%).

Table 3(a) shows the results in terms of acute toxicity, and Tables 3(b) and 3(c) show the impact of age, dose to tumor bed, ENI, and AHT on acute gastrointestinal and genitourinary toxicity, respectively. Only a trend between ENI and gastrointestinal toxicity (*P* = 0.072) has been observed. For acute genitourinary toxicity a trend was observed for both ENI (0.071) and AHT (*P* = 0.05), with a higher incidence (G ≥ 2: 24.3%) in patients treated with LH-RH analogue. However, on multivariate analysis a trend was confirmed only for AHT (ENI: odds ratio: 1.941, CI 95%: 0.567–6.650, and *P*: 0.291; AHT: odds ratio: 1.961, CI 95%: 0.928–4.146, and *P*: 0.078).

Table 4(a) shows the results in terms of late toxicity, and Table 4(b) shows the impact of age, dose to tumor bed, ENI, and AHT on late gastrointestinal and genitourinary toxicity. None of these factors showed a significant correlation with late toxicity. However, it may be noted that none of the patients who received a dose of 64.8 Gy showed grade > 1 late toxicity. Also AHT duration (6 months versus 24 months) did not show a correlation with late toxicity (data not shown).

At the last observation, 1 patient had local recurrence (0.8%), 6 patients had distant metastases (4.9), and 4 patients died (3.3%); in 2 cases, death was due to PCa (1.6%). Actuarial 5-year BDSF was 90.2%.

## 4. Discussion

To improve the results of standard postoperative RT, a phase I/II based on the modulation of adjuvant therapy has been designed. Therefore, different doses on different targets, with eventual drug therapy of varying length, were prescribed depending on pathological assessment. This preliminary analysis was designed to assess the toxicity of this treatment.

**Figure 1:**
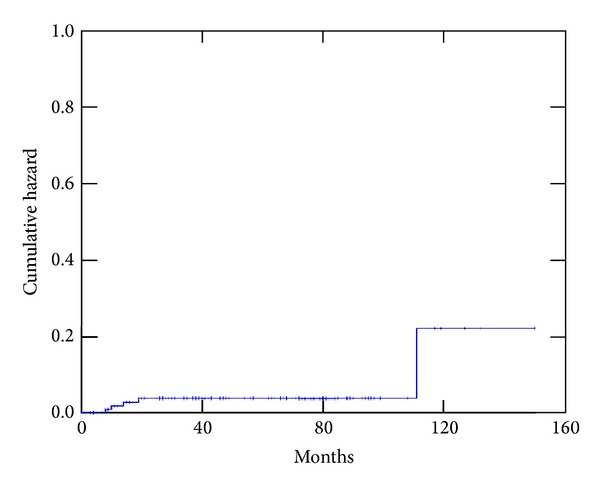
Actuarial cumulative incidence of gastrointestinal (grade > 1) toxicity.

**Figure 2:**
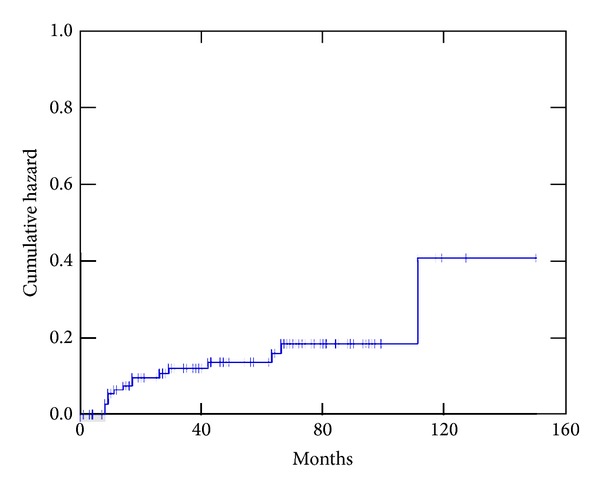
Actuarial cumulative incidence of genitourinary (grade > 1) toxicity.

Overall, the treatment was well tolerated. The incidence of grade 3 acute toxicity was only 2.4% and 3.3% for genitourinary and gastrointestinal toxicity, respectively (see Figures 1 and 2). No patient had late grade 3 gastrointestinal toxicity, and the actuarial 5-year cumulative incidence of grade 3 genitourinary toxicity was 5.8%.

The study has some obvious limitations. The analysis of the correlation between the different parameters of treatment with toxicity is limited by the low number of patients in the different subgroups and the duration of follow-up (median 50.6 months) may be too short for an accurate assessment of late toxicity. It should be noted, however, that 80% of cases of late gastrointestinal toxicity of grade ≥ 2 and 75% of cases of late genitourinary toxicity of grade ≥ 2 occurred in the first 3 years of follow-up. Another problem is that the potential link between comorbidity and toxicity has not been examined. Even the side effects caused by AHT were not analyzed. Finally, another limitation is related to the inhomogeneity of the prescribed AHT. However, the use in a group of patients of antiandrogen therapy was justified by the desire to avoid the side effects of androgen deprivation therapy in patients potentially suffering from the side effects of RP and RT. Second, the results of the RTOG 96-01 trial showed that in patients, with locally advanced disease, bicalutamide 150 mg adjuvant to postoperative radiotherapy demonstrates significant clinical benefits in terms of overall survival, disease-free survival, and BDFS compared with RT alone [27].

Even considering these limitations, there were no significant correlations between dose and ENI with the radiation-induced toxicity. The use of the UDT with prone positioning of the patient and of the 3D technique may at least partially explain the lack of gastrointestinal toxicity in patients who received ENI or a higher dose to the tumor bed.

Table 5 shows the results of our study in comparison with those of the randomized trials. This comparison is not easy because in those studies acute toxicity was not recorded and in two studies the used scale of toxicity was not specified [4, 5]. However, it can be observed that, despite the use of a higher dose and of ENI in 58% of patients, gastrointestinal G2 toxicity (3.7%) was similar to that reported in the randomized studies (1.4%–3.3%). Even the late genitourinary G2 toxicity (12.7%) was in the range (2%–21.3%) recorded in two randomized studies [3, 4]. Even the overall (gastrointestinal and genitourinary) grade 3 late toxicity (5.8%) is comparable with the results (5.3%) of the EORTC study [4]. Also, in this case, the low toxicity despite higher dose and ENI can find an explanation in the use of 3D technique, of UDT, and of slightly lower dose fractionation (1.8 Gy/fraction versus 2.0 Gy/fraction).

Table 6 shows the results of our study compared with those of recent studies on high-dose postoperative RT [24–26]. It is possible to observe that acute gastrointestinal G2 toxicity (10.6%) was similar to the results reported by Nath and coworkers and less than that recorded by van Praet and colleagues (42%). However, the latter prescribed a higher dose both to the tumor bed (75 Gy) and to the pelvic lymph nodes (54 Gy). Similar results are those concerning late gastrointestinal G2 toxicity. Even in this case our results (3.7%) are similar to those of the study of Nath and colleagues (2%) and lower than those recorded by van Praet and collaborators (25%), again probably due to the different doses administered in this latest study.

Acute genitourinary G2 toxicity (10%) was similar to that reported in the study by Nath and colleagues (14%) and lower than that recorded by Cozzarini and coworkers (19%), probably due to the use of 2D technique in a group of patients, and less than that recorded by van Praet and collaborators (35%), again probably due to the different prescribed doses. Similar results were recorded for late genitourinary G2 toxicity (13%), again similar to that of Nath and colleagues (16%) and lower than that of Cozzarini and coworkers (23.9%) and van Praet and collaborators (36%). Again these differences can be explained by the different technique used by Cozzarini and by the different doses prescribed by van Praet.

In terms of late grade 3 genitourinary toxicity, our results (6%) are in the range of those reported in other studies (2–12%). Finally, unlike our study, in the study of van Praet and colleagues a negative impact on the toxicity of ENI was registered. Even in this case, the explanation may come from the different dose used in that study (54 Gy) compared to ours (45 Gy).

An improvement of the results in terms of toxicity may arise in the future by the use of intensity-modulated techniques. For example, a series of our parallel studies showed that the use of postoperative IMRT significantly reduces rectum and bladder irradiation compared to 3D RT [28]. In addition, hypofractionated high-dose IMRT delivered with simultaneous integrated boost (SIB) enables reduction of the overall treatment time, with an acute toxicity profile which compares favourably with that of conventionally fractionated high-dose 3D RT [29, 30].

The positive results of our study may also depend on the use of ENI. This finding confirms a previous observation on the improvement of biochemical recurrence-free survival in patients with high risk PCa undergoing prostate bed plus nodal irradiation after RP [31].

In conclusion, a modulated and intensified adjuvant treatment in PCa was feasible in this phase I/II trial. A further period of observation can provide a complete assessment of late toxicity and confirm the positive results in terms of BDSF.

## Figures and Tables

**Table 1:** Prescribed treatment based on patients/tumor characteristics.

Treatment modulation	Patient/tumor characteristics
Higher dose (70.2 Gy) to the tumor bed	(i) Positive resection margin (ii) Perineural infiltration (iii) Postoperative PSA > 0.2 ng/mL

ENI	(i) pN1 (ii) Lymph node risk > 15%∗ and <10 resected lymph nodes (iii) Gleason score > 7

Short-term (6 months) AHT	(i) pT > 2 (ii) Gleason score = 7

Long-term (24 months) AHT	(i) pN1 (ii) Preoperative PSA > 20 ng/mL (iii) Gleason score > 7

*Based on Roach 3rd [22]. ENI: elective nodal irradiation; AHT: adjuvant hormonal therapy.

**Table 2:** Patients and treatment characteristics.

	Number	%
All patients	123	100
Age (median, range), years	64, 46–78	
pT		
1c	0	0
2a	1	0.8
2b	2	1.6
2c	14	11.4
3a	61	49.6
3b	41	33.3
4	4	3.3
pN		
0	79	64.2
1	18	14.6
*X*	26	21.1
Perineural infiltration		
No	47	38.2
Yes	76	61.8
PSA before surgery (median, range), *μ*g/L	8.8, 0.4–55.0	
PSA after surgery (median, range), *μ*g/L	0.06, 0.01–0.90	
Histopathologic grade, Gleason score		
2–6	23	18.7
7	69	56.1
8–10	31	25.2
Lymphadenectomy		
No	26	21.1
Yes	97	78.9
Interval surgery-radiotherapy (median, range), months	4 (2–9)	
Radiotherapy dose to prostatic bed, Gy		
64.8	18	14.6
70.2	105	85.4
Elective nodal irradiation		
No	52	42.3
Yes	71	57.7
Adjuvant hormonal therapy		
No	38	30.9
Bicalutamide	48	39.0
LH-RH analogue	37	30.1

**Table:** (a)

	Grade				
	0	1	2	3	4
Gastrointestinal	64 (52.0%)	43 (35.0%)	13 (10.6%)	3 (2.4%)	0 (0.0%)
Genitourinary	60 (48.8%)	47 (38.2%)	12 (9.8%)	4 (3.3%)	0 (0.0%)

**Table:** (b)

	Number of patients	Grade			Grade		
		0-1	≥2	*P* =	0–2	≥3	*P* =
Age							
≤65 years	72	60 (83.3%)	12 (16.7%)	0.152	70 (97.2%)	2 (2.8%)	0.772
>65 years	51	47 (92.2%)	4 (7.8%)		50 (98.0%)	1 (2.9%)	
Dose							
64.8 Gy	18	14 (77.8%)	4 (22.2%)	0.185	18 (100.0%)	0 (0.0%)	0.619
70.2 Gy	105	93 (88.6%)	12 (11.4%)		102 (97.1%)	3 (2.9%)	
ENI							
No	52	48 (92.3%)	4 (7.7%)	0.072	52 (100.0%)	0 (0.0%)	0.189
Yes	71	59 (83.1%)	12 (16.9%)		68 (95.8%)	3 (4.2%)	
AHT							
No	38	34 (89.5%)	4 (10.5%)	0.758	37 (97.4%)	1 (2.6%)	0.276
Bicalutamide	48	42 (87.5%)	6 (12.5%)		48 (100.0%)	0 (0.0%)	
LH-RH agonist	37	31 (83.8%)	6 (16.2%)		35 (94.6%)	2 (5.4%)	

ENI: elective nodal irradiation; AHT: adjuvant hormonal therapy.

**Table:** (c)

	Number of patients	Grade			Grade		
		0-1	≥2	*P* =	0–2	≥3	*P* =
Age							
≤65 years	72	62 (86.1%)	10 (13.9%)	0.730	69 (95.8%)	3 (4.2%)	0.497
>65 years	51	45 (88.2%)	6 (11.8%)		50 (98.0%)	1 (2.0%)	
Dose							
64.8 Gy	18	17 (94.4%)	1 (5.6%)	0.278	18 (100.0%)	0 (0.0%)	0.527
70.2 Gy	105	90 (85.7%)	15 (14.3%)		101 (96.2%)	4 (3.8%)	
ENI							
No	52	48 (92.3%)	4 (7.7%)	0.071	50 (96.2%)	2 (3.8%)	0.566
Yes	71	59 (83.1%)	12 (16.9%)		69 (97.2%)	2 (2.8%)	
AHT							
No	38	35 (92.1%)	3 (7.9%)	0.050	37 (97.4%)	1 (2.6%)	0.264
Bicalutamide	48	44 (91.7%)	4 (8.3%)		45 (93.8%)	3 (6.3%)	
LH-RH agonist	37	28 (75.7%)	9 (24.3%)		37 (100.0%)	0 (0.0%)	

ENI: elective nodal irradiation; AHT: adjuvant hormonal therapy.

**Table:** (a)

Toxicity	Grade			
	1	2	3	4
Gastrointestinal	85.4%	96.3%	100.0%	100.0%
Genitourinary	76.5%	87.3%	94.2%	100.0%

**Table:** (b)

Toxicity	Age (years)			Dose (Gy)			ENI			AHT			
	≤65	>65	*P* =	64.8	70.2	*P* =	No	Yes	*P* =	No	BIC	LRA	*P* =
GI ≥ 2	95.2	97.9	0.538	100	95.7	0.348	97.7	95.3	0.425	97.1	94.8	97.2	0.849
GU ≥ 2	86.3	89.0	0.584	100	85.2	0.097	85.2	88.4	0.634	87.7	90.6	85.0	0.893
GU ≥ 3	94.0	95.4	0.781	100	93.2	0.356	93.1	95.4	0.243	95.8	97.7	90.3	0.380

BIC: bicalutamide; ENI: elective nodal irradiation; AHT: adjuvant hormonal therapy; LRA: LH-RH agonists; and ys: years.

**Table 5:** Results (toxicity) comparison with randomized studies.

Study	Number of pts	Adjuvant therapy	Toxicity scores	Acute toxicity		Late toxicity	
				GI	GU	GI	GU
Thompson et al., 2006 [5]	214	RT: 60–64 Gy (2 Gy/fraction) to prostatic fossa and periprostatic tissue	NR	NR	NR	Proctitis and/or rectal bleeding: 3.3% ∗	Urethral stricture: 17.8%; total urinary incontinence: 6.5% ∗

Wiegel et al., 2009 [3] (ARO 96-02/AUO AP 09/95)	114	RT: 60 Gy (2 Gy/fraction) to prostatic fossa and region of seminal vesicles with 1 cm margin	Acute: RTOG Late: RTOG-EORTC	NR	NR	G2: 1.4% G3: 0% †	G2: 2%; G3: 0.7%; urethral stricture: 1.4% †

Bolla et al., 2012 [4] (EORTC 22911)	502	RT: 50 Gy (2 Gy/fraction) to prostatic fossa and region of seminal vesicles and periprostatic area + 10 Gy to prostatic fossa	NR	NR	NR	G ≥ 2: 2.5% ‡	G ≥ 2: 21.3% ‡
						Late GI-GU G ≥ 1: 70.8%	
						Late GI-GU G3: 5.3%	
						Late GI-GU G4: 0%	
						‡	

Present series	123	RT: 64.8–70.2 Gy (1.8 Gy/fraction) to prostatic fossa and region of seminal vesicles with 1 cm margin ± ENI, 45 Gy ± AHT	Acute: RTOG Late: RTOG-EORTC	G3: 2.4% G4: 0.0%	G3: 3.3% G4: 0.0%	G ≥ 2: 3.7% G ≥ 3: 0.0% G ≥ 4: 0.0% §	G ≥ 2: 12.7% G ≥ 3: 5.8% G ≥ 4: 0.0% §

ENI: elective nodal irradiation; GI: gastrointestinal; GU: genitourinary; AHT: adjuvant hormonal therapy; NR: not reported; pts: patients; ∗: crude (median follow-up: 10.6 years); †: crude (median follow-up: 53.7 months); ‡: 10-year cumulative incidence; and §: 5-year actuarial cumulative incidence.

**Table 6:** Results (toxicity) comparison with nonrandomized studies using high-dose radiotherapy.

Study	Number of pts	Adjuvant therapy	RT technique	Toxicity scores	Acute toxicity		Late toxicity		Notes
					GI	GU	GI	GU	
Nath et al., 2010 [24]	50	RT (median dose: 68 Gy)	IMRT-IGRT	CTC 3.0	G2: 8% G3: 0%	G2: 14% G3: 0%	G2: 2% ∗	G2: 16% G3: 2% ∗	IMRT-IGRT may reduce RT-induced toxicity

Cozzarini et al., 2012 [25]	556	RT (median dose: 70.2 Gy) ± ENI	2D or 3D	CTC 3.0	NR	G2: 19% G3: 8%	NR	G2: 23.9% G3: 12% †	Younger and hypertensive pts: higher rate of severe GU late sequelae

van Praet et al., 2013 [26]	48 (pN1)	RT (75 Gy to prostate bed + ENI: 54 Gy) + ADT	IMAT	In-house developed scale	G2: 42% G3: 0%	G2: 35% G3: 4%	G2: 25% G3: 0% ‡	G2: 36% G3: 7% G4: 2% ‡	Acute and late GI toxicity higher following ENI

Present series	123	RT (64.8–70.2 Gy to prostate bed ± ENI) ± AHT	3D CRT	Acute: RTOG Late: RTOG-EORTC	G2: 10.6% G3: 2.4% G4: 0%	G2: 9.8% G3: 3.3% G4: 0%	G ≥ 2: 3.7% G ≥ 3: 0% G ≥ 4: 0% §	G ≥ 2: 12.7% G ≥ 3: 5.8% G ≥ 4: 0% §	No significant effect on toxicity by age, dose, ENI, and AHT

3D CRT: 3-dimensional conformal radiation therapy; ADT: androgen deprivation therapy; AHT: adjuvant hormonal therapy; ENI: elective nodal irradiation; GI: gastrointestinal; GU: genitourinary; IGRT: image guided radiation therapy; IMAT: intensity-modulated arc therapy; IMRT: intensity-modulated radiation therapy; pts: patients; ∗: crude, median follow-up: 24 months; †: 8-year risk; ‡: crude (only patients with ≥12-month follow-up); and §: 5-year actuarial cumulative incidence.
